# Single-variant genome-wide association study and regional heritability mapping of protein efficiency and performance traits in Large White pigs

**DOI:** 10.1186/s12711-025-00993-z

**Published:** 2025-08-14

**Authors:** Esther Oluwada Ewaoluwagbemiga, Audald Lloret-Villas, Adéla Nosková, Hubert Pausch, Claudia Kasper

**Affiliations:** 1 Agroscope, Animal GenoPhenomics, Tioleyre 4, 1725 Posieux, Switzerland; 2 Animal Genomics, Department of Environmental Systems Science, ETH Zurich, Universitätstrasse 2, 8092 Zurich, Switzerland

## Abstract

**Background:**

Improvement of protein efficiency (PE) is a key factor for a sustainable pig production, as nitrogen excretion contributes substantially to environmental pollution. Protein efficiency has been shown to be heritable and genetically correlated with performance traits such as feed conversion ratio (FCR) and average daily feed intake (ADFI). This study aimed to identify genomic regions associated with these traits through single-variant genome-wide association studies (GWAS) and regional heritability mapping (RHM) using whole-genome sequence variants from low-pass sequencing of more than 1000 Swiss Large White pigs.

**Results:**

Genomic heritability estimates using ~ 15 million variants were moderate to high, ranging from 0.33 to 0.47. GWAS did not identify significant variants for PE and FCR, but identified 45 variants at suggestive significance levels for ADFI on chromosome 1 and one for ADG on chromosome 14. Similarly, RHM detected no significant regions for PE and FCR, but five suggestive regions for ADFI (chromosome 1) and one for ADG (chromosome 14). However, by combining leading signals from GWAS and RHM, i.e. overlapping leading variants and significant regions, we highlighted putative candidate genes for PE, including *PHYKPL, COL23A1, PPFIBP2, GVIN1, SYT9, RBMXL2, ZNF215,* and olfactory receptor genes.

**Conclusions:**

Combining GWAS and RHM allowed us to identify genomic regions that may influence PE and production traits. Our apparent difficulty in detecting significant regions for these traits probably reflects the relatively small sample size, differences in genetic architecture across study designs and experimental conditions, and that polymorphisms explaining large proportions of the trait variation may not segregate in this population. Nevertheless, we identified plausible functional candidate genes in the highlighted regions, including those involved in nutrient sensing, the urea cycle, and metabolic pathways, in particular IGF1-insulin, and that have previously been reported to be associated with nitrogen metabolism in cattle and with muscle and adipose tissue metabolism and feed intake in pigs. We also highlighted a range of noncoding RNAs. Their targets and roles in gene regulation should be further investigated in this context.

**Supplementary Information:**

The online version contains supplementary material available at 10.1186/s12711-025-00993-z.

## Background

As demand for meat continues to grow worldwide, the need to produce livestock more sustainably is becoming increasingly urgent due to the environmental impact of intensive farming. A key pollutant in livestock production is nitrogen, which forms harmful compounds such as nitrate, ammonia, and nitrous oxide [1–3]. Although pigs are more protein efficient than cattle [4], with nitrogen excretion of 70–80% in beef and dairy production [5–7], only approximately 50% of the dietary protein consumed by pigs is excreted as waste [8, 9]. Undigested proteins are excreted in faeces, while absorbed amino acids that are not used for growth or maintenance are eliminated in urine [10]. The latter is an environmental concern because the nitrogen in urine is in a volatile form [11, 12]. The percentage of nitrogen in urine is influenced by the fibre content of the diet, its fermentability, nitrogen digestibility and the pig's growth and maintenance requirements, and ranges from 48.6% on a pectin-enriched diet [12] to 79.3% on a grain-based diet [11]. Methods such as reducing dietary nitrogen [13, 14] and selection to increase protein efficiency (PE; the proportion of total dietary protein intake retained in the carcass) in pigs [15] have been proposed to reduce the contribution of animal-based food production to environmental pollution. Heritability estimates ranging from 0.16 to 0.54 have been recently reported for PE and related traits (e.g., nitrogen digestible coefficient, protein deposition), depending on the breed, fattening phase, and diet type [16]. The significant genetic variance and moderate to high heritability (h^2^) estimates for PE and nitrogen use efficiency from these studies indicate that these traits can be genetically improved and thus presents promising targets towards a more sustainable pig production through reduced nitrogen excretion. Performance traits such as feed conversion ratio (FCR), average daily feed intake (ADFI), and average daily gain (ADG) are also important, considering their economic and environmental impacts. Several studies have reported moderate heritabilities for these traits [15, 17] and genetic correlations among them in pigs [16] and other species. Genetic correlations (± SE) among these traits have been reported in Large White pigs, including −0.55 ± 0.14 between PE and FCR [15], −0.24 ± 0.10 between the digestibility coefficient of nitrogen (DCN) and FCR of [18], −0.53 ± 0.14 between PE and ADFI [15], −0.53 ± 0.13 between DCN and ADFI of [18], −0.19 ± 0.19 between PE and ADG [15], and −0.15 ± 0.17 between DCN and ADG [18] have been previously reported. A strong negative genetic correlation between the digestibility of protein (DP) and FCR has also been observed in broilers [19].

Genome-wide association studies (GWASs) in pigs have reported loci associated with several important traits, such as meat quality [20], performance [21, 22], carcass [23], body composition [24], and efficiency-related traits [25]. However, despite the environmental importance of nutrient efficiency traits, such as PE and nitrogen excretion, to date, only Shirali et al. [26] and Schmid et al. [27] have identified genomic regions associated with nitrogen-excretion traits and nitrogen use efficiency, respectively, in pigs. Shirali et al. [26] used 315 pigs from Piétrain grand-sires and grand-dams from a three-way cross, with pigs genotyped for 88 microsatellite markers on 10 (of 18) chromosomes [26]. Their study identified three quantitative trait loci (QTL) associated with total nitrogen excretion throughout the 60–140 kg body weight (BW) growth period on chromosomes (SSC) 2, 4, and 7 [26]. Three additional QTL were found for average daily nitrogen excretion for the same growth period, on SSC 6, 9, and 14 [26]. However, the study by Shirali et al. [26] was limited by the use of a small number of genetic markers that were unevenly distributed across the genome and covered only 10 chromosomes. In addition, the design of their study was fundamentally different from ours, as it was a linkage study using an F₂ cross, which is primarily designed to identify broad genetic differences between founder lines rather than to explain genetic variation within a population, and is therefore less suitable for fine-mapping or detecting within-breed associations. Schmid et al. [27] reported significant SNPs for nitrogen use efficiency in German Landrace × Piétrain crosses on SSC 5 and 13 during fattening phase 1 (40 kg BW) and on SSC 6 only during fattening phase 2 (60 kg BW). Although this study included a considerably larger number of markers (over 48,000 SNPs) than Shirali et al. [26], the sample size was relatively limited (N = 502). Furthermore, the nitrogen use efficiency phenotype was predicted from N-balance data and blood metabolites using a prediction equation derived from detailed phenotyping of only 48 animals [28], which was then applied to a larger set of 508 animals in [27]. It is also noteworthy that both these studies [26, 27] involved Piétrain crossbreds, which likely differ significantly in genetic background from the Large White animals used in this study (see [16] for discussion).

As FCR, ADG, and ADFI have either direct or indirect impacts on efficiency and production costs, a number of studies have identified QTL for FCR, ADG, and ADFI [27, 29–32]. The majority of these QTL were found in Duroc and Landrace pigs, with only a few QTL identified in Large White pigs [33]. However, although FCR and RFI have been reported to be correlated with PE by Ewaoluwagbemiga et al. [15] and Saintilan et al. [17], it has been suggested that selecting for improved FCR and RFI is less efficient at reducing nutrient excretion than selecting directly for the nutrient efficiency trait (e.g., PE) itself in poultry [34], a notion that is likely extendable to other species.

Complex traits are typically influenced by many genes (i.e., are polygenic), with many genetic variants having too small effect sizes to be detected at the Bonferroni-corrected or false-discovery rate (FDR) threshold of single-variant GWAS [35, 36]. This makes it difficult to identify associated variants, particularly for traits for which limited sample sizes are available. Besides GWAS, regional heritability mapping (RHM) offers an alternative approach to identify genotype–phenotype associations [37, 38]. RHM divides the genome into windows of a certain number of variants. Subsequently, a genomic relationship matrix (GRM) is computed using all variants in each region, and the variance of the trait explained by each region is estimated [39]. RHM identifies regions that contribute to the genetic variance of a trait by aggregating the effects of multiple variants within a defined region, rather than analyzing them individually [39]. This approach can capture signals from variants with effects that are too small to be detected by GWAS, albeit at the cost of lower resolution because it estimates the heritability of broader genomic regions rather than pinpointing specific variants. By grouping multiple variants, RHM reduces the number of statistical tests required, which allows for less stringent correction for multiple testing. However, RHM's power to detect associations depends on the extent to which genetic variation within the region is collectively influential, and its performance may vary with factors such as regional linkage disequilibrium structure and trait architecture.

A limitation of RHM is that it estimates the contribution of a region to trait variance by modeling it as a random effect, which generally requires larger sample sizes to obtain reliable estimates. This is in contrast to single-variant GWAS, which typically models variants as fixed effects and thus involves simpler parameter estimation. As a result, RHM may be less efficient in smaller datasets or when regional effects are modest. Nevertheless, the RHM approach has been shown to be effective in several studies; for example, Resende et al. [40] detected 26 QTL associated with 7 traits in eucalyptus using RHM, compared to only 13 QTL identified by single-variant GWAS. Similarly, Sutera et al. [41] found 5 QTL associated with fat percentage in sheep using RHM. In this study, we used RHM alongside single-variant GWAS with the aim of providing a complementary analysis that can further clarify results.

The aim of this study was therefore to investigate the genetic basis of PE and performance traits in Swiss Large White pigs. This was achieved by estimating the genomic heritability and performing GWAS and RHM using low-pass sequence data.

## Methods

### Animals and phenotypes

We analysed a total of 1036 Swiss Large White pigs, which were previously included in several nutrition experiments and one genetic study. The Swiss Large White dam line used in our study has been selected for high fertility with good piglet rearing ability, but also for production, albeit to a lesser extent than the Swiss Large White sire line. Together, fattening performance, feed conversion, and lean meat content account for around 30% of the selection index and meat quality accounts for around 14% of the selection index. To produce crossbred fattening pigs in practice, a crossbred sow (Large White dam line × Swiss Landrace) and a boar from the Large White sire line are used, the latter being primarily selected for meat quality, fattening performance, FCR, and lean meat content (37, 26, 19 and 8% relative weights in the selection index, respectively).

The data set used in this study is described in detail in Ewaoluwagbemiga et al. [15]. Briefly, data from 7 experiments, which were carried out at Agroscope Posieux in Switzerland, were combined. Piglets were weaned at an average age of 27 ± 2 days after birth by removing the sow and were fed a standard starter diet with crude protein levels following the Swiss feeding recommendations for pigs [42]. At 22.3 (± 1.6) kg, pigs were placed in pens equipped with automatic feeders (single-spaced automatic feeder stations with individual pig recognition system by Schauer Maschinenfabrik GmbH & Co. KG, Prambachkirchen, Austria) and stayed in this pen until slaughter. Pigs had ad libitum access to isocaloric diets that differed in crude protein or fibre content. The majority of pigs (853) received a diet that contained 80% of the crude protein and digestible essential amino acids content of the control diets in both grower and finisher phase, while 16 pigs received a standard (control) diet in the grower and the protein-reduced diet only in the finisher phase. The three control groups had no reduction in dietary crude protein and their diets were mainly formulated according to recommendations for pigs [42]. One control diet (154 pigs) conformed to the recommendations for all nutrients. Two control diets were used in an organic pig production trial and contained 95 to 100% organic ingredients (32 pigs). A third 100% organic control diet (16 pigs) was formulated to contain 6% more crude fibre than the other control diets. In all experiments, pigs were fed a grower diet from approximately 20 to 60 kg live BW and a finisher diet from 60 kg to slaughter at 100 kg. Most pigs (840) were slaughtered at about 100 kg BW, 32 pigs at 40 kg, 57 at 60 kg, 50 at 80 kg, 53 at 120 kg, and 40 at 140 kg live BW. Pigs that were slaughtered at 120 and 140 kg were fed another, specially formulated, finisher diet from 100 to 140 kg [43]. Most of the animals were females (492) or castrated males (469), and only 110 were intact males. To account for the heterogeneity in the data, we included the diet type (i.e. treatment), slaughter weight, and sex as fixed effects in the model.

Every week, pigs were weighed individually, and, once a pig reached a live BW of approximately 20 kg, it was allocated to a grower-finisher pen, and the experimental treatments were started. This was done until a maximum number of 12 (or 24 or 48) pigs per pen was reached (depending on the pen layout; at least 1 m^2^ per pig and at most 12 pigs/feeder). Pigs remained in their pen until slaughter. The automatic feeder recorded all visits and feed consumption per visit, from which the total feed intake of each pig was calculated. The protein content of feed was monitored during production by near-infrared spectroscopy for each 500 kg batch. To obtain more accurate data on feed composition at the time of consumption, a sample was taken from each automatic feeder station each week, and the crude protein content was determined by wet-chemistry methods.

### Phenotype data

The phenotypes were derived as reported in Ewaoluwagbemiga et al. [15]. Total and average daily feed intake (ADFI) were recorded, and average daily gain (ADG) and the feed conversion ratio (FCR) were calculated as follows:

1 $$ADG = \frac{{live BW \left( {kg} \right) slaughter - live BW \left( {kg} \right) start}}{{age \left( {days} \right) slaughter - age \left( {days} \right) start}}$$

2 $$FCR = \frac{ADFI}{{ADG}}$$

where $live BW \left(kg\right) slaughter$ and $age \left(days\right) slaughter$ are the live pre-slaughter body weight in kg and the age in days at slaughter, respectively, and $live BW \left(kg\right) start$ and $age \left(days\right) start$ are the body weight in kg and the age in days at the start of the grower phase, respectively. To measure PE, the left carcass half of each pig, including the whole head and tail, was scanned with a dual-energy X-ray absorptiometry (DXA; GE Lunar i-DXA, GE Medical Systems, Glattbrugg, Switzerland) to determine the lean tissue content, which was used in the equation of Kasper et al. [44] to estimate the protein content retained in the carcass;

3 $$CP_{carcass} \left( g \right) = - 482.745 + 0.23 \left( {Lean_{DXA} \left( g \right)\, \times \,P} \right)$$

where ${}carcass\left(g\right)$ is the crude protein content of the carcass in g, $Lean_{DXA}\left( g \right)$ is the lean meat content obtained with DXA in g, and $P$ is the proportion of the left cold carcass (also includes the whole head and tail) to the total cold carcass weight. This method of estimating carcass protein content using DXA yields a highly precise and accurate phenotype with an R^2^ between 0.983 and 0.998 [44, 45]. Unlike other techniques, such as estimating carcass protein using semi-mechanistic models [46] or determining protein digestibility from faeces with near-infrared spectroscopy [18], this approach allows calculating PE as protein retained over protein intake without relying on assumptions and, therefore, likely preserves individual differences in the trait. Protein efficiency was calculated as;

4 $$\textit{protein efficiency} = \frac{{CP_{carcass} \left( g \right) slaughter - CP_{carcass} \left( g \right) start }}{{CP_{feed} intake \left( g \right)}}$$

where $C{P}_{carcass} (g) slaughter$ is the crude protein content of carcass at slaughter, ${CP}_{carcass} (g) start$ is the crude protein content of carcass at the start of the experiment, and ${CP}_{feed} intake (g)$ is the crude protein feed intake. The crude protein content of pigs at the start of the experiment $({CP}_{carcass} \left(g\right)start)$ was estimated from a sample of 38 piglets (12 females, 12 castrated males and 14 entire males). These piglets were slaughtered at an average of 20.98 ± 1.85 kg BW in a previous experiment and their carcass protein content was chemically determined [43]. The average protein content per kg carcass for each sex (female, entire male, castrated male) was used to estimate ${CP}_{carcass} (g) start$ for the pigs by multiplying the BW of pigs when they entered the experiment (i.e., at approximately 20 kg body weight) with the protein content per kg carcass of piglet, as previously determined from the 38 piglets [43].

### Genotype data and imputation

The sampled pigs were genotyped on three different platforms using DNA extracted from blood: (i) 258 pigs were genotyped at 600 K using the Axiom Porcine Genotyping array; (ii) 297 pigs were sequenced at an intended read depth of 4 × ; and (iii) 492 pigs were sequenced at an intended read depth of 1 × .

#### Imputation of array data

The array genotyping data was imputed to whole genome sequence level using Beagle (v 4.1) [47] based on a reference panel of 421 pigs (Landrace and Large White, including the 297 pigs in (ii)) that were sequenced at a coverage ranging between 4 × and 37.5 × . The imputation accuracy was 0.92 (R^2^) [33, 48]. The imputed array data finally contained 29,469,425 autosomal variants (biallelic SNPs and indels).

#### Filtering and imputation of 4 × sequencing data

We estimated the average realized coverage at 4.5 × ± 0.9 × (calculated as the amount of raw data obtained divided by the approximate pig genome size of 2.8 GB). Filtering and alignment to the reference genome were carried out as described in [49]. In brief, raw reads with a phred quality score below 15 for more than 15% of the bases were removed using the fastp software (version 0.19.4) with default parameter settings [50]. The filtered reads were aligned to the Sus Scrofa 11.1 assembly [51] using the MEM-algorithm of the Burrows-Wheeler Alignment (BWA) software (version 0.7.17) [52, 53] and the alignment quality and coverage depth were assessed. Duplicate reads and reads with a mapping quality ≤ 10 were removed. Imputation of sporadically missing genotypes in the variant files was done using Beagle (v 4.1) [47] with an imputation accuracy of 0.98 [48]. The 4 × sequenced data contained 30,179,303 variants.

#### Filtering and imputation of 1 × sequencing data

The 1 × data was imputed using Gencove’s loimpute pipeline v0.1.5 [54] using 414 publicly available pig sequence data, with an imputation accuracy of 0.97. The data contained 45,100,556 autosomal variants (including 13,361,070 non-variant sites) and the realized coverage after mapping and variant calling was 0.61 × .

#### Merged variant call format file

Eight pigs without phenotypes and three pigs with a mismatch between pedigree and genomic-based relationships were excluded from further analyses. PLINK (v1.9) [55, 56] was used to merge the (imputed) genotypes from the three different genotyping approaches based on their physical positions according to the reference genome (*Sus Scrofa 11.1*). After merging, there were 23,171,650 intersecting biallelic variants (including indels) and 1036 individuals with genotypes.

### Single-variant genome-wide association study

Prior to GWAS, we tested for outliers in the phenotypes and removed individuals with phenotypes not in the range of µ ± 3σ. This resulted in 1025, 1033, 1034, and 1024 individuals remaining for PE, ADG, ADFI, and FCR, respectively. For each trait, we removed variants with minor allele frequency (MAF) < 5% and variants that deviated from Hardy–Weinberg equilibrium (P < 0.0001). After quality control, 15,269,953, 15,192,400, 15,200,584, and 15,220,328 variants were included for PE, ADFI, ADG, and FCR, respectively.

Residuals of linear mixed-effects models in the lme4 package [57] in R software v 4.2. [58] adjusted for systematic environmental effects were used as phenotypes in single-variant GWAS for each trait. The environmental effects included the fixed effects from a model selection step prior to estimating genetic parameters as described in Ewaoluwagbemiga et al. [15], including year (factor variable), dietary treatment (factor variable), sex (factor variable; castrates, females and males), slaughter weight, ambient temperature in the barn at the start of the experiment, slaughter age, interaction of slaughter weight and sex, interaction of treatment and sex, interaction of treatment and slaughter age, and interaction of year and slaughter age. However, it should be noted that while the model accounts for these fixed effects, the genetic architecture of the traits may vary between the different experimental conditions that pigs were exposed to. The model we used in our analyses assumes variant effects to be identical for all these conditions.

The single-variant GWAS was performed with GCTA using the fastGWA method [59], where SNP effects were tested one at a time using a linear mixed effects model approach, incorporating the genomic relationship matrix (GRM) to account for relatedness in the sampled population. The linear mixed effects model fitted to the data was

5 $${\varvec{y}} = \mu + b{\varvec{m}} + {\varvec{a}} + {\varvec{e}}$$

where ${\varvec{y}}$ is a vector of residuals of phenotypes corrected for systematic environmental effects; $\mu$ is the overall mean; $b$ is the allele substitution effect; ${\varvec{m}}$ is the vector of marker genotypes, coded as 0, 1, and 2; ${\varvec{a}}$ is the vector of random polygenic effects following the distribution ${\varvec{a}}\sim N(0, {\varvec{G}}{\sigma }_{a}^{2})$, ${\varvec{G}}$ is the GRM and ${\sigma }_{a}^{2}$ is the additive genetic variance; ${\varvec{e}}$ is the vector of residual effects assumed to follow ${\varvec{e}}\boldsymbol{ }\sim N(0,{\varvec{I}}{\sigma }_{e}^{2})$**,** where ${\varvec{I}}$ is the identity matrix and ${\sigma }_{e}^{2}$ is the residual variance.

The heritability of a specific variant was estimated in GCTA using an approach analogous to that described below for regional heritability mapping. Two GRMs were used, one for the entire genome and the other restricted to the variant of interest. Heritability was calculated as the proportion of phenotypic variance explained by the variant, i.e. the variance attributed to the variant divided by the total phenotypic variance (the sum of the variance attributed to the genomic variant, the genome-wide variance and the residual variance). The statistical significance of the heritability estimate was assessed using a likelihood ratio test, as described below.

We adopted two approaches to obtain meaningful significance thresholds for single-variant GWAS, which balanced the need to correct for multiple testing with the fact that several tested variants are in strong LD and thus not independent. First, we used an empirical procedure to obtain a distribution of the test statistic (in this case the p-value) under the null hypothesis. To do so, we followed the procedure for permutation testing described in van den Berg et al. [60], which was based on Churchill and Doerge [61]. Specifically, we ran 1000 GWASs for each of the traits using the same model and the same method as described above, but with the phenotype vector (i.e. the residuals) randomly shuffled in each run. For each iteration, we recorded the smallest p-value and we defined the experiment-wise critical value as the -log_10_ of the p-value at the 95th percentile [61]. Second, we applied a Bonferroni-corrected significance threshold at an alpha level of 0.05, based on the number of sufficiently independent loci derived from the typical LD-decay. In commercial pig lines, markers at distances of 100–150 kb have been shown to have an average r^2^ of about 0.3 [62]. Based on this, we chose a slightly more conservative approach and pruned the data using Plink 1.9 (– indep-pairwise 100 10 0.4) and used the number of remaining variants to calculate the threshold. We inspected the quantile–quantile (QQ) plots for the inflation of small p-values, which could indicate population stratification. The genomic inflation factor was used to investigate a potential violation of the distribution assumptions of non-associated variants, which can arise due to population stratification [63]. Genomic inflation was calculated as the median of the observed chi-squared test statistics divided by the distribution of the expected chi-square test statistics under the null hypothesis of no association.

### Regional heritability mapping

Regional heritability mapping was performed using the GCTA software [59]. For this analysis, each chromosome (SSC1 to SSC18) was divided into several windows that contained 5000 variants each and that overlapped by 2500 variants. Windows at the end of the chromosomes contained on average 3837.5 variants, but never less than 2533. Subsequently, the genomic variance was estimated for each of the around 5230 regions of the genome (their exact number depended on the trait). The linear mixed effects model below was used to test the effect of all variants within each genomic region, which included the random regional genomic effect and the random genomic effect of the rest of the genome, including the specific region:

6 $${\varvec{y}} = {\varvec{Zk}} + {\varvec{Zu}} + {\varvec{e}}$$

where ${\varvec{y}}$ is a vector of the residuals of phenotypes corrected for environmental effects, as indicated above for the single-variant GWAS; ${\varvec{Z}}$ is the design matrix for the random effects; ${\varvec{k}}$ is the vector of random regional additive genomic effects following the distribution ${\varvec{k}}\sim N\left(0,{{\varvec{G}}}_{{\varvec{k}}}{\sigma }_{k}^{2}\right),$ and ${\varvec{u}}$ is the vector of random polygenic effects of all variants following the distribution $\mathbf{u}\sim N\left(0,{{\varvec{G}}}_{{\varvec{u}}}{\sigma }_{u}^{2}\right)$. Here, ${{\varvec{G}}}_{{\varvec{k}}}$ is the regional GRM, ${\sigma }_{k}^{2}$ is the regional variance, ${{\varvec{G}}}_{{\varvec{u}}}$ is the whole genome GRM, and ${\sigma }_{u}^{2}$ is the genome additive genetic variance. Regional and whole genome heritability were estimated as ${h}_{k}^{2}=\frac{{\sigma }_{k}^{2}}{{\sigma }_{p}^{2}}$ and ${h}_{u}^{2}=\frac{{\sigma }_{u}^{2}}{{\sigma }_{p}^{2}}$, respectively, where ${\sigma }_{p}^{2}$ is the sum of the regional variance (${\sigma }_{k}^{2}$), whole genome variance (${\sigma }_{u}^{2}$), and residual variance (${\sigma }_{e}^{2}$). Statistical significance of the variance of a region was tested using the likelihood ratio test (LRT), which compares the log likelihood of the full model (including regional and whole genome variance) with the reduced model (including only the whole genome variance). This was done by specifying the –reml-lrt 1 option in GCTA, which gives the LRT and p-value of the random regional additive genomic effect.

To identify significant and suggestive regions, two different thresholds were defined. It should be noted that the thresholds for statistical significance differ between single-variant GWAS and RHM in our study. Here, for the genome-wide significance threshold, Bonferroni correction for multiple testing was applied at an alpha level of 0.05 for the around 5230 regions, divided by 2 to account for the overlap. Instead of an LD-pruned threshold as used for single-variant GWAS, we set a suggestive threshold for RHM following the procedure described in [41]. Briefly, the suggestive threshold implies that, for every genome scan, one false positive is allowed for [41]. The thresholds applied in the current study for RHM were thus at p-values of 1.94 × 10^–5^ (−log_10_(p) = 4.71) and 3.89 × 10^–4^ (−log_10_(p) = 3.413) for the genome-wide 5% significance and the suggestive thresholds, respectively.

### Identification of genes in significant regions

After performing GWAS and RHM, we identified genes located within the significant and/or suggestive regions. These genes were identified using the pig reference genome (*Sus Scrofa 11.1*) on the genome data viewer [64], using the NCBI Sus scrofa Annotation Release 106 and Ensembl release 110 (Accession NC_010444.4). The biological functions of candidate genes were found using the Gene Ontology database [65, 66]. The platforms AnimalQTLdb [67] pigGTEx [68] were used to retrieve additional information on previously identified QTL and molecular QTL, such as expression QTL (eQTL), QTL for long noncoding RNA expression (lncQTL), enhancer QTL (enQTL), and alternative splicing QTL (sQTL). We restrict reporting to such QTL for tissues of interest for our traits, including muscle, liver, tissues of the immune system and the gastrointestinal tract, and the brain.

## Results

### Single-variant GWAS

**Fig. 1:**
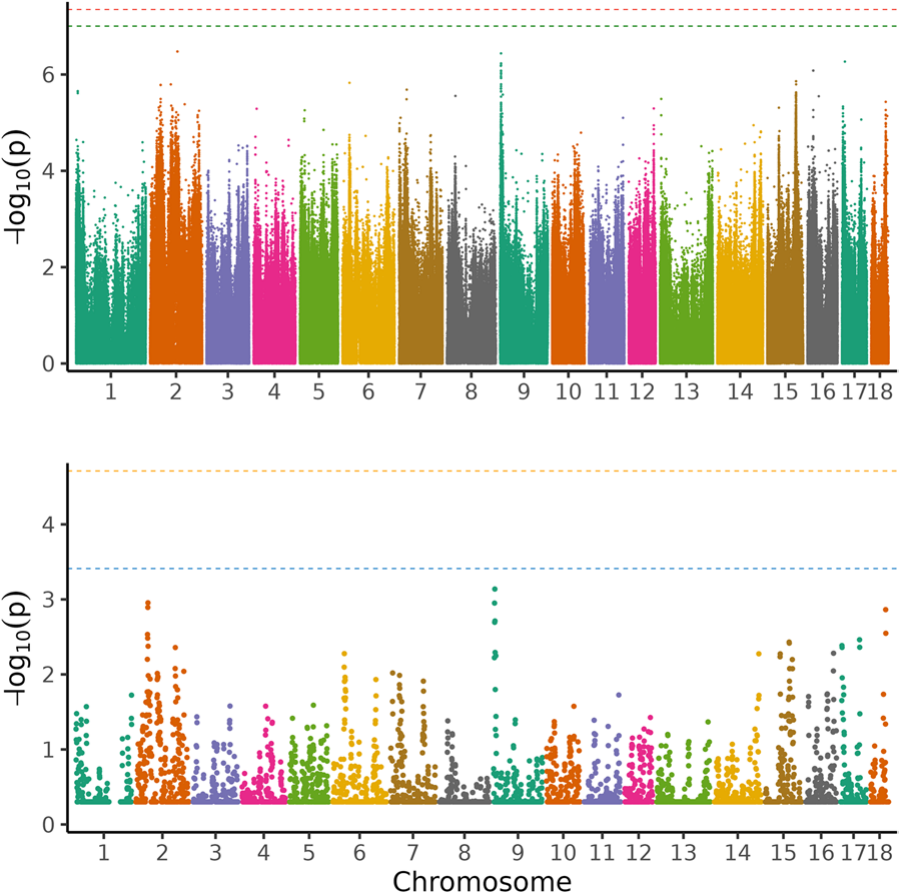
Manhattan plot of the single-variant genome-wide association analysis (above) and regional heritability mapping (below) of protein efficiency. The *x*-axis and the *y*-axis represent the chromosomes and the observed −log_10_(*P*-value), respectively. The red line is the threshold obtained by permutation, the green line in the Manhattan plot is the Bonferroni-corrected significance threshold at an alpha level of 0.05 (based on the number of sufficiently independent loci). In the lower panel, the orange line is the Bonferroni-corrected significance threshold at an alpha level of 0.05 (based on the number of unique windows tested) and the blue line is the suggestive threshold

**Fig. 2:**
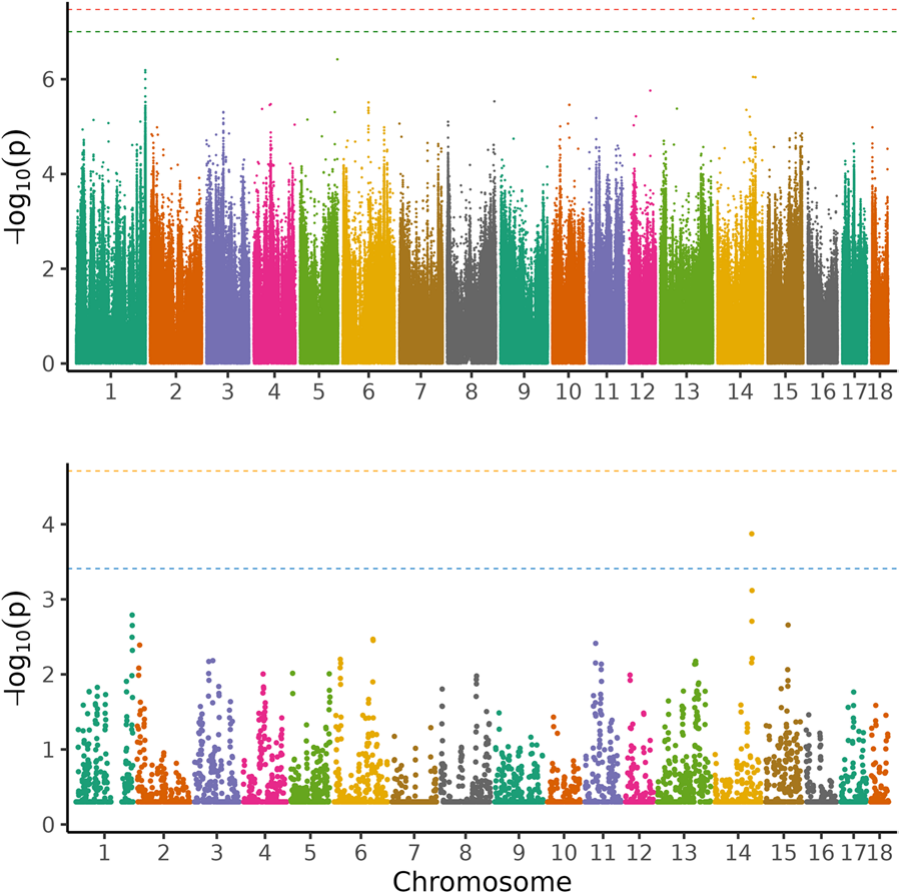
Manhattan plot of the single-variant genome-wide association analysis (above) and regional heritability mapping (below) of feed conversion ratio. The *x*-axis and the *y*-axis represent the chromosomes and the observed −log_10_(*P*-value), respectively. The red line is the threshold obtained by permutation, the green line in the Manhattan plot is the Bonferroni-corrected significance threshold at an alpha level of 0.05 (based on the number of sufficiently independent loci). In the lower panel, the orange line is the Bonferroni-corrected significance threshold at an alpha level of 0.05 (based on the number of unique windows tested) and the blue line is the suggestive threshold

**Fig. 3:**
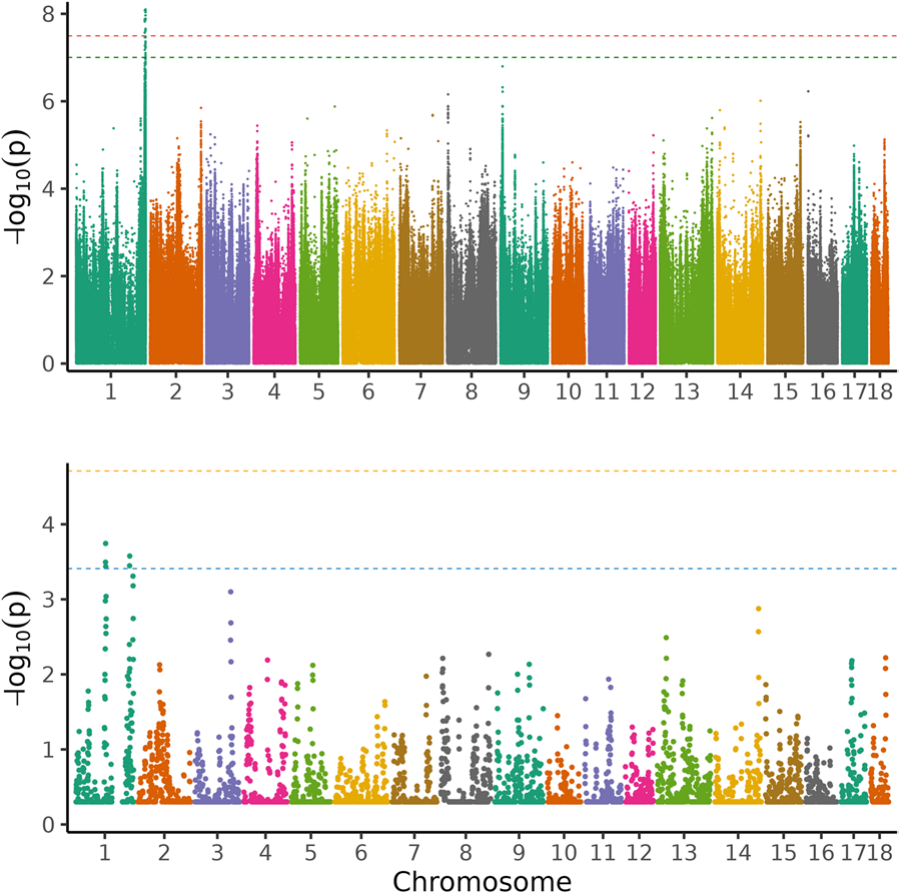
Manhattan plot of the single-variant genome-wide association analysis (above) and regional heritability mapping (below) of average daily gain. The *x*-axis and the *y*-axis represent the chromosomes and the observed −log_10_(*P*-value), respectively. The red line is the threshold obtained by permutation, the green line in the Manhattan plot is the Bonferroni-corrected significance threshold at an alpha level of 0.05 (based on the number of sufficiently independent loci). In the lower panel, the orange line is the Bonferroni-corrected significance threshold at an alpha level of 0.05 (based on the number of unique windows tested) and the blue line is the suggestive threshold

**Fig. 4:**
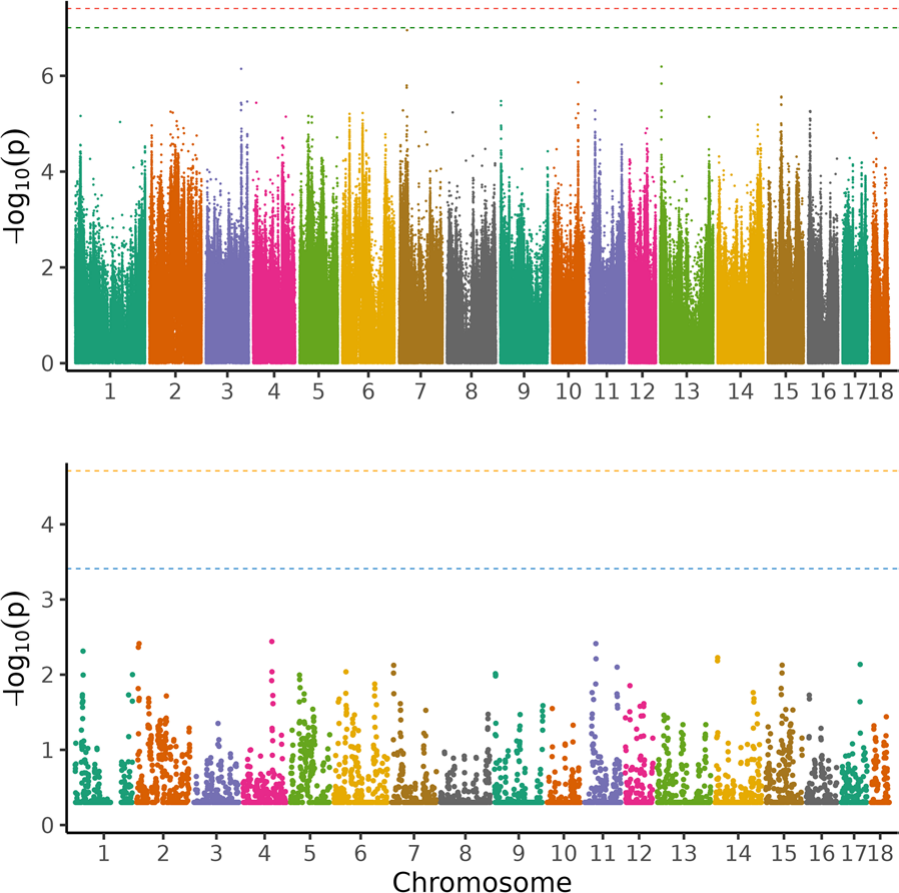
Manhattan plot of the single-variant genome-wide association analysis (above) and regional heritability mapping (below) of average daily feed intake. The *x*-axis and the *y*-axis represent the chromosomes and the observed −log_10_(*P*-value), respectively. The red line is the threshold obtained by permutation, the green line in the Manhattan plot is the Bonferroni-corrected significance threshold at an alpha level of 0.05 (based on the number of sufficiently independent loci). In the lower panel, the orange line is the Bonferroni-corrected significance threshold at an alpha level of 0.05 (based on the number of unique windows tested) and the blue line is the suggestive threshold

**Fig. 5:**
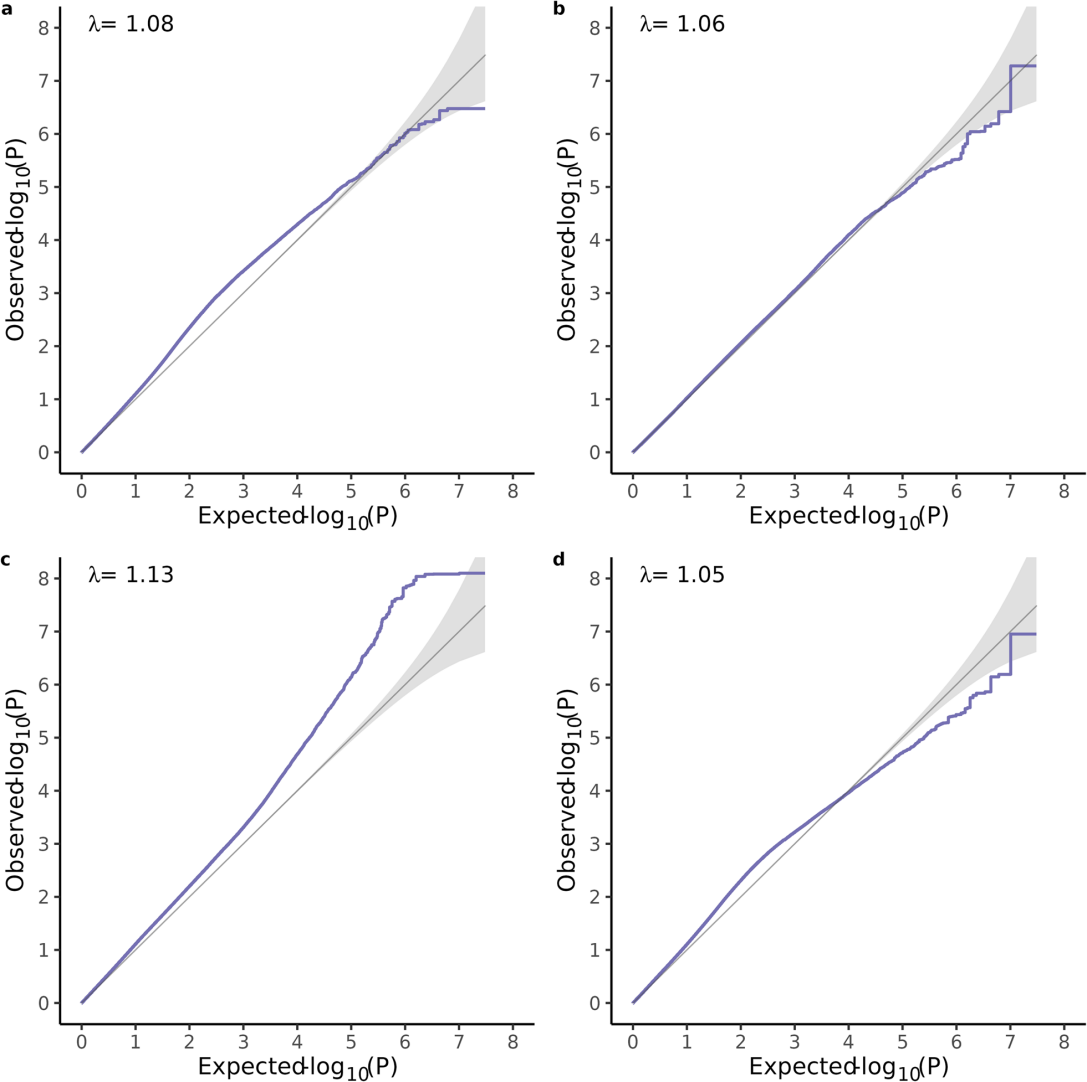
Quantile–Quantile plots of single-variant GWAS. **a** protein efficiency, **b** Average daily gain (ADG), **c** Average daily feed intake (ADFI) and, **d** feed conversion ratio (FCR). λ is the genomic inflation factor

The Bonferroni thresholds based on LD pruned genotype data for all traits, at the alpha level of 0.05, were 9.90 × 10^–8^, 9.93 × 10^–8^, 9.94 × 10^–8^, 9.91 × 10^–8^ for PE, ADG, ADFI, and FCR, respectively. The thresholds obtained with the permutation approach were 4.47 × 10^–8^, 3.56 × 10^–8^, 3.19 × 10^–8^, and 3.93 × 10^–8^, respectively, for PE, ADG, ADFI, and FCR. No significant variants were found at either threshold for PE (Fig. 1, Table 1; see Additional file 1, Table S1). For ADG, one variant on SSC14 passed the LD-pruned threshold (Fig. 2, Table 2; see Additional file 2, Table S2), for ADFI, 26 variants on SSC1 (25 SNPs and 1 indel) passed the permutation threshold, and 19 further variants on SSC1 (15 SNPs and 4 indels) also passed the LD-pruned threshold (Fig. 3, Table 3; see Additional file 3, Table S3). No variants passed either threshold for FCR (Fig. 4, Table 4; see Additional file 4, Table S4). The heritability (± SE) of the suggestive variant for ADG was h^2^ = 0.02 ± 0.03, around 7% of the total genomic heritability, and the heritability of the 45 variants for ADFI together was h^2^ = 0.06 ± 0.04, around 14% of the total genomic heritability. The QQ plots of the GWAS analyses (Fig. 5a–d) and the genomic inflation factor, which was approximately 1 for all traits (PE = 1.08, ADG = 1.06, ADFI = 1.13, FCR = 1.05), suggested that population stratification was sufficiently accounted for by the GRM. Although no or few suggestive associations were found for PE and FCR, the genomic heritability for traits ranged from 0.33 to 0.47 using all available variants (Table 5), with ADG having a slightly higher genomic heritability (0.47) than pedigree-based heritability (0.45).

**Table 1:** Top 5 variants from single-variant GWAS for protein efficiency with their position on the chromosome in base pairs, the gene they are located in, the variant ID and the alleles

Rank	SSC	Position	A1	A2	AF1	Beta	SE	P	Gene	Variant	Alleles	Nearby genes
1	2	79,836,276	T	C	0.057	−0.014	0.003	3.33 × 10^–07^	*COL23A1*	rs690223530	C/T	*CLK4* and *PHYKPL*
2	2	79,836,286	A	G	0.057	−0.014	0.003	3.33 × 10^–07^	*COL23A1*	rs701405971	G/A	*CLK4* and *PHYKPL*
3	9	2,619,789	C	T	0.173	0.009	0.002	3.64 × 10^–07^	–	rs324093704	T/C	ENSSSCG00000038122^a^, *ZNF215* and several olfactory receptor genes
4	17	7,290,544	CA	C	0.118	−0.010	0.002	5.40 × 10^–07^	*TRIML1*	–	–	*TRIML2*
5	9	2,712,088	C	A	0.172	0.009	0.002	5.89 × 10^–07^	–	rs1108123956	C/A	Several olfactory receptor genes

Nearby genes (within a range of 100 kb up- and downstream) are also listed. Variants did not reach the permutation (4.47 × 10^–8^) nor the LD-pruned Bonferroni threshold (9.90 × 10^–8^). *A1* reference allele, *A2* alternative allele, *AF1* allele frequency of reference allele, Beta allele substitution effect with standard error (SE) and p-value (P)

^a^pseudogene

**Table 2:** Top 5 variants from single-variant GWAS for average daily gain with their position on the chromosome in base pairs, the gene they are located in, the variant ID and the alleles

Rank	SSC	Position	A1	A2	AF1	Beta	SE	P	Gene	Variant	Alleles	Nearby genes
1	14	110,924,112	G	A	0.054	0.032	0.006	5.23 × 10^–08^	CUTC	rs337699748	A/G	COX15, ENTBD7 and ABCC2
2	5	102,186,460	C	T	0.115	−0.020	0.004	3.80 × 10^–07^	SYT1	rs710124645	C/T	ENSSSCG00000049637
3	1	272,909,885	AAG	A	0.276	−0.014	0.003	6.43 × 10^–07^	lncRNA^b^	–	–	OBP2B, ENSSSCG00000043049, GBGT1, RAGLDS and ABO, SURF6, RPLA7, MED22, SURF4, STKLD1
4	1	272,909,895	C	CA	0.277	−0.014	0.003	7.16 × 10^–07^	lncRNA^b^	–	–	
5	14	110,176,234	TA	T	0.075	0.0244	0.005	8.96 × 10^–07^	HPSE2	rs699719557^a^	A/T	–

Nearby genes (within a range of 100 kb up- and downstream) are also listed. Variants ranked 2–5 did not reach the genome-wide (3.56 × 10^–8^) nor the suggestive threshold (9.93 × 10^–8^), but the first-ranking variant reached the suggestive threshold

*A1* reference allele, *A2* alternative allele, *AF1* allele frequency of reference allele, Beta allele substitution effect with standard error (SE) and p-value (P)

^b^ENSSSCG00000062610

^a^variant located 1 bp upstream in Genome Data Viewer

**Table 3:** Significant (in bold) and suggestive (in italics) variants from single-variant GWAS for average daily feed intake with their position on SSC1 in base pairs, the gene they are located in, the variant ID and the alleles

Rank	Position	A1	A2	AF1	Beta	SE	P	Gene	Variant	Alleles	Nearby genes
1	273,496,587	T	G	0.278	0.049	0.008	7.95 × 10^–09^	–	rs342474793	G/T	VAV2 and BRD3
2	273,496,457	A	G	0.275	0.049	0.008	8.25 × 10^–09^	–	rs333113518	G/A	VAV2 and BRD3
3	273,496,470	T	G	0.275	0.049	0.008	8.25 × 10^–09^	–	rs340291882	G/T	VAV2 and BRD3
4	273,496,486	C	T	0.275	0.049	0.008	8.25 × 10^–09^	–	rs323875909	T/C	VAV2 and BRD3
5	270,982,720	C	A	0.100	0.069	0.012	8.32 × 10^–09^	–	rs712027998	A/C	FIBCD1 and LAMC3
6	273,496,625	C	T	0.278	0.049	0.008	8.34 × 10^–09^	–	rs326407596	T/C	VAV2 and BRD3
7	273,496,743	G	A	0.275	0.048	0.008	9.16 × 10^–09^	–	rs325682162	A/G	VAV2 and BRD3
8	273,496,749	G	A	0.275	0.048	0.008	9.16 × 10^–09^	–	rs339310280	A/G	VAV2 and BRD3
9	273,496,759	A	G	0.275	0.048	0.008	9.16 × 10^–09^	–	rs320598057	G/A	VAV2 and BRD3
10	273,513,647	C	G	0.248	0.051	0.009	1.08 × 10^–08^	BRD3	rs321803809	G/C	VAV2 and WDR5
11	273,496,721	C	G	0.275	0.048	0.008	1.29 × 10^–08^	–	rs344411671	G/C	VAV2 and BRD3
12	270,589,733	C	T	0.172	0.055	0.010	1.33 × 10^–08^	–	rs322817388	T/C	ASS1
13	273,473,392	T	C	0.247	0.050	0.009	1.38 × 10^–08^	VAV2	rs333938622	C/T	SARDH and BRD3OS
14	270,599,545	G	A	0.161	0.056	0.010	1.39 × 10^–08^	–	rs326035908	A/G	ASS1
15	270,589,693	A	G	0.172	0.055	0.010	1.50 × 10^–08^	–	rs335988374	G/A	ASS1
16	270,589,695	T	G	0.172	0.055	0.010	1.50 × 10^–08^	–	rs340768103	G/T	ASS1
17	273,481,971	A	G	0.245	0.050	0.009	2.20 × 10^–08^	–	rs323301985	G/A	VAV2
18	273,473,590	C	T	0.243	0.049	0.009	2.38 × 10^–08^	VAV2	rs335502076	T/C	SARDH and BRD3OS
19	273,473,601	C	T	0.243	0.049	0.009	2.38 × 10^–08^	VAV2	rs319552596	T/C	SARDH and BRD3OS
20	273,473,603	A	C	0.243	0.049	0.009	2.38 × 10^–08^	VAV2	rs713897779	C/A	SARDH and BRD3OS
21	273,473,613	C	CG	0.243	0.049	0.009	2.38 × 10^–08^	VAV2	rs793814574	CG/C	SARDH and BRD3OS
22	273,487,853	T	C	0.250	0.049	0.009	2.43 × 10^–08^	–	rs335311042	C/T	VAV2 and BRD3
23	272,839,507	C	T	0.472	-0.041	0.007	2.47 × 10^–08^	LOC110256503^a^	rs3473060690	T/C	GBGT1
24	270,599,467	T	C	0.161	0.055	0.010	2.69 × 10^–08^	–	rs699921403	C/T	ASS1
25	270,599,489	A	G	0.161	0.055	0.010	2.69 × 10^–08^	–	rs713018859	G/A	ASS1
26	270,599,528	A	G	0.161	0.055	0.010	2.69 × 10^–08^	–	rs706359608	G/A	ASS1
27	273,517,030	T	C	0.249	0.049	0.009	3.32 × 10^–08^	BRD3	rs333674056	C/T	VAV2 and WDR5
28	270,599,666	T	TATATAC	0.162	0.055	0.010	3.43 × 10^–08^	–	rs713961016	TATATAC/T	ASS1
29	273,481,384	A	G	0.248	0.049	0.009	3.47 × 10^–08^	–	rs318478034	G/A	VAV2 and BRD3
30	273,445,392	T	C	0.250	0.049	0.009	4.29 × 10^–08^	VAV2	rs325919278	C/T	BRD3
31	273,496,990	A	G	0.273	0.047	0.009	4.65 × 10^–08^	–	rs340187248	G/A	VAV2 and BRD3
32	273,484,075	C	CAGGGA	0.247	0.048	0.009	4.70 × 10^–08^	–	rs786847961	CAGGGA/C	VAV2 and BRD3
33	273,469,880	T	C	0.249	0.049	0.009	4.85 × 10^–08^	VAV2	rs328126130	C/T	BRD3
34	270,982,734	G	A	0.101	0.065	0.012	5.34 × 10^–08^	–	rs1109265720	A/G	FIBCD1 and LAMC3
35	273,474,175	AG	A	0.244	0.048	0.009	5.49 × 10^–08^	VAV2	rs1107705322	A/G	BRD3
36	273,545,902	T	C	0.242	0.048	0.009	5.54 × 10^–08^	–	rs320931279	C/T	VAV2 and WDR5
37	273,445,528	T	C	0.251	0.048	0.009	5.70 × 10^–08^	VAV2	rs329357351	T/C	BRD3
38	273,445,540	C	T	0.251	0.048	0.009	5.70 × 10^–08^	VAV2	rs338673132	C/T	BRD3
39	273,496,268	AATAG	A	0.270	0.046	0.009	5.76 × 10^–08^	–	rs706632263	A/AATAG	VAV2 and BRD3
40	273,519,510	A	G	0.249	0.048	0.009	6.02 × 10^–08^	BRD3	rs691215421	G/A	VAV2 and WDR5
41	270,589,753	T	C	0.171	0.053	0.010	6.67 × 10^–08^	–	rs332410580	C/T	ASS1
42	273,473,786	A	G	0.242	0.048	0.009	7.87 × 10^–08^	VAV2	rs344778327	G/A	BRD3
43	273,470,949	C	T	0.248	0.048	0.009	8.76 × 10^–08^	VAV2	rs324192941	T/C	BRD3
44	273,484,432	G	C	0.251	0.047	0.009	8.79 × 10^–08^	–	rs329390136	C/G	VAV2 and BRD3
45	273,473,274	A	G	0.246	0.047	0.009	9.74 × 10^–08^	VAV2	rs325075572	G/A	BRD3

Nearby genes (within a range of 100 kb up- and downstream) are also listed. Variants ranked 1–26 reached the permutation threshold (3.19 × 10^–8^), and those ranked 27–45 the LD-pruned Bonferroni threshold (9.94 × 10^–8^). *A1* reference allele, *A2* alternative allele, *AF1* allele frequency of reference allele, Beta allele substitution effect with standard error (SE) and p-value (P)

^a^non-coding RNA

**Table 4:** Top 5 variants from single-variant GWAS for feed conversion ratio with their position on the chromosome in base pairs, the gene they are located in, the variant ID and the alleles

Rank	SSC	Position	A1	A2	AF1	Beta	SE	P	Gene	Variant	Alleles	Nearby genes
1	7	22,869,310	T	C	0.132	0.052	0.010	1.12 × 10^–07^	SLA-1 and LOC100515902^a^	rs703864294	C/T	TRIM26 and SLA-5, SLA-2, SLA-3
2	13	5,032,974	C	A	0.387	−0.032	0.006	6.42 × 10^–07^	ENSSSCG00000043505	rs325347387	A/C	ENSSSCG00000052924, ENSSSCG00000055104 and ENSSSCG00000043579
3	3	109,132,075	G	A	0.092	0.055	0.011	7.14 × 10^–07^	–	rs329329516	A/G	ENSSSCG00000059546, LOC106509860^b^, LBH and ENSSSCG00000052409^c^, YPEL5, LOC100621247^b^, ENSSSCG00000030917, ENSSSCG00000059894
4	10	55,303,764	A	T	0.215	0.036	0.007	1.37 × 10^–06^	MALRD1	rs336494865	T/A	–
5	13	5,032,996	C	T	0.382	-0.031	0.006	1.45 × 10^–06^	ENSSSCG00000043505	rs338820653	T/C	ENSSSCG00000052924, ENSSSCG00000055104 and ENSSSCG00000043579

Nearby genes (within a range of 100 kb up- and downstream) are also listed. None of the variants reached the permutation (3.93 × 10^–8^) or the LD-pruned Bonferroni threshold (9.91 × 10^–8^). *A1* reference allele, *A2* alternative allele, *AF1* allele frequency of reference allele, Beta allele substitution effect with standard error (SE) and p-value (P)

^a^HLA class I histocompatibility antigen, A-11 alpha chain-like

^b^pseudogene

^c^lncRNA

**Table 5:** Descriptive statistics and pedigree and genomic heritability estimates for protein efficiency and performance traits

Trait	*N*	Mean ± SD	Min–Max	h^2^_ped_ (SE)	h^2^_geno_ (SE)
PE	1025	0.39 ± 0.03	0.28–0.49	0.54 (0.10)	0.42 (0.05)
ADG	1033	0.85 ± 0.11	0.51–1.20	0.45 (0.11)	0.47 (0.04)
ADFI	1034	2.26 ± 0.31	1.30–3.14	0.53 (0.12)	0.42 (0.05)
FCR	1024	2.68 ± 0.21	1.99–3.77	0.39 (0.12)	0.33 (0.04)

*PE* Protein efficiency, *ADG* average daily gain, *ADFI* average daily feed intake, *FCR* feed conversion ratio

### Regional heritability mapping

In total, around 5230 genomic regions were subjected to RHM for each trait. No region was found significant at the genome-wide level for any trait (Figs. 1, 2, 3, and 4) and no region reached the suggestive threshold for PE (Fig. 1; see Additional file 5, Table S5). There was one region on SSC14 that passed the suggestive threshold for ADG, with a regional heritability of 0.15 (Fig. 2; see Additional file 6, Table S6). Five regions reached the suggestive threshold for ADFI on SSC1 (Fig. 3; see Additional file 7, Table S7), including three overlapping windows (635,000_640,000, 637,500_642,500, and 640,000_645,000 bp) and another two windows that overlapped (1,155,000_1,160,000 and 1,157,500_1,162,500 bp). The regional heritabilities for these five windows were rather low (0.06 to 0.1). For FCR, there were no regions that passed either threshold (Fig. 4; see Additional file 8, Table S8) and regional heritabilities were low across the genome (0.008 to 0.04).

### Genomic regions based on RHM

For PE, the two methods seemed to agree quite well and confirmed each other (Fig. 1). The top five variants in single-variant GWAS were on SSC2, SSC9 and SSC17. In RHM, the windows that were closest to the suggestive threshold (−log_10_(P) = 3.41) were found on SSC2 and SSC9. However, the highest-ranking variants in single-variant GWAS on SSC2 were not included in the highest-ranking windows of RHM, but three other variants in the GWAS top 50 (ranked 26–28) were included in window 205,000_210,000, which was ranked 10 in RHM (Tables S1 and S5). This window overlapped with two others on SSC2 in the top ten windows (205,000_210,000, 207,500_212,500 and 210,000_215,000; including 10,000 variants), spanning the region from 30,144,793 to 32,170,603 bp. The highest-ranking variant on SSC9 was also not found in a RHM window in the top 50, but 17 variants on SSC9 (ranking 5–33) were present in the top-ranking window in RHM (20,000_25,000; see Additional file 5, Table S5). Two adjacent windows were top ranking on SSC9 (15,000_20,000 and 20,000_25,000), which encompassed 10,000 variants and spanned the region from 2,013,927 to 2,730,410 bp. The suggestive variant on SSC14 in single-variant GWAS for ADG was not present in a top 50 window in RHM and the suggestive window on SSC14 (637,500_642,500) did not contain any variant in the top 50 (Fig. 2; see Additional file 2, Table S2 and Additional file 6, Table S6). However, three other variants among the top 10 in single-variant GWAS on SSC14 were found in the adjacent window ranking 18th (635,000_640,000; both windows ranging from 117,072,833 to 118,516,125 bp). All 19 variants on SSC1 in the top 50 were present in two overlapping windows in RHM (1,227,500_1,232,500 and 1,230,000_1,235,300; from 272,754,902 to 273,355,069 bp, spanning 7500 variants). Two variants on SSC1 in the top 5 were found in 2 overlapping windows among the top 5 (Table 2; see Additional file 6, Table S6). The variant on SSC5 ranked 2nd in single-variant GWAS was present in the 30th ranking window in RHM (662,500_667,500). All 45 variants that passed one of the thresholds in single-variant GWAS for ADFI belonged to two overlapping windows in RHM (1,232,500_1,237,500 and 1,235,000_1,240,000) on SSC1 within the region of 273,495,531–273,690,809 bp (Fig. 3; see Additional file 3, Table S3 and Additional file 6, Table S6). In general, the pattern for FCR was less consistent than for the other traits, with no overlap of top 5 variants and windows (Fig. 4; see Additional file 4, Table S4 and Additional file 8, Table S8). For instance, one variant on SSC7 was rather close to the suggestive threshold (−log_10_(P) = 7.00) in single-variant GWAS, but it was not present in any window in the RHM top 50.

### Genes related to genomic variants and regional heritability mapping windows

The first two variants from single-variant GWAS for PE were located on SSC2at 79,836,276 bp and 79,836,286 bp (Table 1). These variants were found within the *COL23A1* gene and were approximately 13 to 36 kb downstream from the start and end of *CLK4* and 977 bp upstream from *PHYKPL*. The third and the fifth ranking variants were located on SSC9 at 2,619,789 bp and 2,619,789 bp, respectively. Both variants were within an intergenic region and flanked by four different olfactory receptor genes. The fourth lead variant was on SSC17 in the *TRIML1* gene. Genes located in the leading windows in RHM for PE are shown in Additional file 9, Table S9. Several protein coding genes, lncRNAs and one pseudogene, were located in the two adjacent windows on SSC9, such as *PPFIBP2, OLFML1*, *SYT9, RBMXL2, NLRP14, ZNF215,* and 16 olfactory receptor genes. The three overlapping windows on SSC2 contained protein coding genes such as *MPPED2*, *ARL14EP*, *FSHB*, *KCNA4,* and *METTL15*, as well as 25 lncRNAs and 3 pseudogenes. For ADG, the suggestive region identified by single-variant GWAS on SSC14 at 110,924,112 bp was situated in the *CUTC* gene (Table 2). The second-ranked variant on SSC5 at 102,186,460 bp was in the *SYT1* gene. Variants ranked 3 and 4 were found within an intergenic region on SSC1 between *OBP2B* and *ABO*. The *HPSE2* gene on SSC14 harboured the fifth-ranking variant (110,176,234 bp). The suggestive variant was not found in a window that reached the significance or suggestive threshold in RHM, but the suggestive window on SSC14 contained the *SORCS1* gene, 2 noncoding RNAs, 6 lncRNAs, and one small nucleolar RNA (see Additional file 10, Table S10). Three further variants (ranks 6–8) in single-variant GWAS (see Additional file 2, Table S2) were located within the *SORCS1* gene, all within one RHM window (635,000_640,000) spanning from 117,072,833 to 118,069,574 bp on SSC14. Several variants on SSC1 were included in overlapping windows (1,227,500_1,232,500 and 1,230,000_1,235,000) that contained 25 protein coding genes, 1 noncoding RNA, 7 lncRNAs, and 5 small nucleolar RNA (see Additional file 10, Table S10).

Three variants for ADFI on SSC1 identified in single-variant GWAS (one significant and two suggestive) were located in the *BRD3* gene, five significant (located between 273.4733 and273.4736 Mb) and eight suggestive variants in the *VAV2* gene, and one in the uncharacterized LOC110256503, a non-coding RNA, which is 11.58–8.516 kb downstream of the *GBGT1* gene (Table 3). The remaining 19 significant and 9 suggestive variants were located in the vicinity of *VAV2* and *BRD3*, *FIBCD1* and *LAMC3*, *ASS1*, *SARDH* and *BRD3OS* (*BRD3* opposite strand), and *WDR5*. The *VAV2* gene is approximately 8 kb downstream from *SARDH* and 84 kb upstream from *WDR5*. The two RHM windows from 273,498,257 to 273,634,463 bp that contained all significant and suggestive variants also spanned the *BRD3*, *BRD3OS*, and *WDR5* genes, as well as six lncRNA, a snRNA (RNU6ATAC), one miRNA (MIR9812), and another noncoding RNA (see Additional file 11, Table S11). The genes in the three suggestive overlapping RHM windows that ranged from 103,336,199 to 105,376,226 bp and two overlapping windows that ranged from 260,888,411 to 262,071,057 bp on SSC1 harboured 30 protein coding genes (for example *MBD2*, *STARD6*, *TCF4*, *PSMD5,* and *RAB14*), six pseudogenes, 15 lncRNAs, and one snRNA (see Additional file 11, Table S11). The close-to-suggestive variant for FCR on SSC7 was located in the SLA-1 gene and in a HLA class I histocompatibility antigen, A-11 alpha chain-like gene (LOC100515902), near *TRIM26* and *SLA-5, SLA-2*, and *SLA-3*, coding for several Scr-like adapter proteins. Variants that were ranked 2 and 5 on SSC13 were located in ENSSSCG00000043505, with no described genes nearby, and the third and fourth-ranking variants were located in an intergenic region and in the *MALRD1* gene, respectively.

## Discussion

Here, we aimed to identify genomic regions associated with PE, ADG, ADFI, and FCR using both single-variant genome-wide association studies (GWAS) and regional heritability mapping (RHM). We observed differences in the strength of evidence supporting associations between genomic variants or regions and phenotypes across different traits. While we identified several variants and windows at both the significant and suggestive thresholds for ADFI and at the suggestive threshold for ADG, the evidence for variants and windows underlying PE, although present below the threshold in both single-variant GWAS and RHM individually, increased our confidence when the results were integrated. In contrast, this combined approach failed to uncover any clear genomic regions associated with FCR.

### Genes associated with traits

#### Average daily feed intake

We identified 26 significant variants for ADFI (P < 4 × 10^–8^), and a further 19 variants that reached the suggestive threshold (P < 9.9 × 10^–8^), all on SSC1 within the region of from 270 to 273.5 Mb. Our study also found five suggestive regions associated with ADFI located at 103–105 Mb and 260–262 Mb on SSC1. Nosková et al. [33] also detected a QTL associated with ADFI on SSC1 at the 270–272 Mb position in the same population. This chromosomal region also harbours QTL for ADFI in other pig breeds and populations [69–71], suggesting a common ancestral origin of the underlying genetic variants, although independent mutational events in the same region cannot be excluded.

Of the SNPs associated with ADFI, the *VAV2* gene had the largest number of significant variants mapped to it. This gene plays a key role in immunity by preventing bacterial attachment and subsequent uptake into cells [72]. It is also involved in pathways critical for skeletal muscle growth and metabolic regulation via the IGF1-insulin pathway. *VAV2* deficiency in mice led to reduced muscle mass, insulin responsiveness, and ultimately symptoms resembling metabolic syndrome [73].

The *BRD3* gene also contained several ADFI-associated variants. It encodes a protein that influences the expression of other genes by increasing their accessibility to RNA polymerase II for transcription [74] and regulates the expression of several immunity-related genes, thereby constituting an important part of the innate immunity [75]. In pigs, *BRD3* has been shown to be involved in immune response to African swine fever [76] and porcine endogenous retrovirus [77]. The level of methylation of *BRD3* in the jejunum of piglets has been suggested to play a role in gut maturation and adaptation to bacterial colonisation associated with milk feeding [78].

Intergenic variants associated with ADFI were located near *FIBCD1, LAMC3,* and the lncRNA ENSSSCG00000042995. *FIBCD1* encodes a chitin-binding receptor with functions in the innate immune system. It responds to helminth antigens, helping intestinal cells control fungal colonization and shape the composition of the gut microbiome [79]. This role is further supported by findings from a mouse model, where overexpression of *FIBCD1* mitigated chemotherapy-induced weight loss, probably by reducing mucositis and gastrointestinal dysbiosis [80]. In pigs, a SNP close to *FIBCD1* was associated with the lean meat content of ham [81]. The *LAMC3* gene is part of the extracellular matrix pathway (GO: 0031012) and influences tissue development, repair, differentiation, and cell migration [82]. As such, it is implicated in adipose tissue remodelling, inflammation, and insulin resistance associated with obesity [83]. In pigs, its expression in adipose tissue was associated with fatness traits [84] and varied in muscle depending on dietary fat content [84].

*ASS1*, another gene in the vicinity of several variants associated with ADFI in an intergenic region, encodes the urea cycle enzyme argininosuccinate synthetase, which is required to convert neurotoxic ammonia to urea in the liver. Deficiency of this gene is associated with metabolic disorders such as citrullinemia [85], insulin resistance, and diabetes [86] in humans. It contributes to innate immune responses to viral [86] and bacterial infections, with expression changes observed in a porcine liver injury model after lipopolysaccharide challenge [87]. Due to its role in the response to growth hormone process (GO:0060416), *ASS1* is a likely candidate gene for growth and body size in pigs [88, 89]. It has also been proposed as a candidate gene for feed efficiency in pigs, probably as a host genetic effect [89]. *ASS1* expression in newborn piglets responded dynamically to antibiotics and maternal fecal microbiome transplant interventions, influencing alanine biosynthesis and amino acid metabolism [90]. Through the biological process’response to nutrient’ (GO:0007584), this gene may directly regulate feed intake by sensing the nutrient composition of the diet and adapting intake to the body's nutrient requirements. This is further supported by the lower expression observed in the umbilical vein of intrauterine growth-retarded piglets, likely due to reduced fetal nutrient supply [91]. It is also linked to energy production and fat storage via the cellular response to oleic acid process (GO:0071400). Higher *ASS1* expression was observed in the semimembranosus muscle of fatter Pulawska pigs compared to the leaner Polish Landrace [92], and its expression was positively correlated with fat area between the 13th and 14th rib of the longissimus dorsi muscle [93].

We identified a significant variant in the uncharacterised lncRNA LOC110256503 (located between 272,836,566 and 272,843,572 bp on SSC1), which is partially overlapping ENSSSCG00065003108.1 (272,839,760 to 272,843,574 bp). It is located near *GBGT1*, which starts around 8 kb upstream and encodes the Forssman synthase, a member of the ABO gene family, which determines blood group antigens through glycosyltransferase activity. This gene is likely inactive in pigs, as in humans and other species [94, 95], suggesting it is not likely to be the regulatory target of this lncRNA. Given that lncRNAs can regulate the transcription, translation, and metabolism of a wide range of genes and their products, including those distant from their own genomic location [96], detailed investigation is required to identify the regulatory targets of this lncRNA and to determine whether its function is linked to genes associated with traits such as ADFI.

Another intergenic variant for ADFI was located near *WDR5*, 75 kb downstream of the start of *BRD3*. It regulates gene expression through histone modification and has a broad range of functions in development, cell division, signal transduction, vesicular trafficking, cytoskeletal assembly, cell cycle control, and apoptosis [97, 98]. It is the target of a long intergenic noncoding RNA (MSTRG.2530) associated with differences in skeletal muscle growth between Yorkshire and Tibetan pigs [99]. Another ADFI variant (at 270,982,734 bp on SSC1) is located 21 kb upstream of *LAMC3* and 65 kb downstream of the end of *QRFP*, encoding a hypothalamic neuropeptide that is highly evolutionarily conserved. It influences feeding behaviour and glucose homeostasis through G protein-coupled receptors [100, 101]. Administration of the *QRFP* peptide directly into the brain of mice resulted in increased feed intake and foraging behaviours [102]. It has been proposed as a prohormone gene (the precursor molecule of a neuropeptide) in pigs [103] and may underlie the reduced backfat thickness observed in Pietrain compared to other pig breeds [104]. A selection signature related to backfat thickness at the shoulder in Yorkshire pigs harboured this gene [105] and a variant associated with residual feed intake was located near it [89].

The three overlapping suggestive RHM windows (103,336,199–105,376,226 bp) on SSC1 contained protein-coding genes with roles in pig production traits, including *MBD22*, *STARD6*, *RAB27B,* and *TCF4*. *MBD2* is involved in the differentiation of porcine mesenchymal stem cells into adipocytes [106] and regulates intramuscular fat content and fatty acid composition in the pig longissimus dorsi muscle [107]. *STARD6* binds cholesterol and other steroids and plays a role in lipid transport and metabolism [108, 109]. *RAB27B*, a target of the lncRNA MSTRG.28207.43, was significantly upregulated in the colon during heat stress-induced intestinal inflammation in pigs [110]. The transcription factor *TCF4* regulates myogenesis [111] and is involved in intestinal epithelium [112] and muscle development in pigs [113]. The two overlapping suggestive windows on SSC1 at 260,888,411–262,071,057 bp harboured genes with multiple documented roles in pigs, such as *PSMD5*, which was differentially expressed in the blood of piglets from lines differentially selected for RFI [114], and *PHF19*, both of which were found in a selection signature in Indian pigs [115]. The gene *TRAF1*, involved in antiviral response and innate immunity [116], was associated with viral load in PRRS-infected pigs [117]. Another innate immunity-related gene, *C5*, was implicated in differences in monocyte levels in pig blood [118]. *RAB14*, which is involved in regulation of hepatic insulin signalling and glucose metabolism [119], was also found in co-expression networks for skeletal muscle myogenesis across species [120].

#### Average daily gain

For ADG, we identified a suggestive variant in the *CUTC* gene on SSC14. It is an evolutionary conserved copper transporter protein, important for maintaining intracellular copper ion homeostasis [121]. In pigs, Sahana et al. [122] reported an FCR-associated SNP near the *CUTC* gene. In Nelore cattle, this gene harbors an allele-specific expression QTL in the longissimus thoraci muscle that might affect meat quality traits [123]. The suggestive region identified by RHM on SSC14 contained *SORCS1*, which is implicated in obesity-induced type 2 diabetes [124, 125]. It is a sorting receptor that helps direct proteins to their correct locations in cells, and is found in hypothalamic neurons, where it attenuates signaling by *BDNF* (brain‐derived neurotrophic factor), thereby regulating energy homeostasis [126]. The knock-out of *SORCS1* together with *SORCS3* in mice leads to the overproduction of an orexigenic neuropeptide and results in increased food intake, decreased locomotor activity, reduced use of lipids as metabolic fuel, and increased adiposity, despite overall reduced body weight [127]. *SORCS1* was suggested as candidate gene for backfat thickness in pigs [128], for fat deposition in beef cattle across several breeds [128], and for FCR in Pekin ducks [129].

#### Protein efficiency

Neither single-variant GWAS nor RHM yielded significant or suggestive results for PE, but both methods suggested the potential involvement of variants on SSC2 and SSC9. Previously, Shirali et al. [26] found associations on SSC2 and SSC9 for nitrogen excretion traits during the 60–140 kg growth stage, but it is unclear whether these associations were present in the same region as those for PE in our study as they used a different reference genome. The two top-ranking variants on SSC2 in single-variant GWAS were located in *COL23A1*, with the genes *CLK4* and *PHYKPL* nearby (Table 2). *COL23A1* has been associated with meat quality [130] and cholesterol levels in pigs [131] and is differentially methylated in humans with regard to waist-to-hip ratio [132] and body mass index [133]. In men, but not women, this gene is associated with weight gain after weight loss [134]. Both *PHYKPL* and *COL23A1* were implicated in nitrogen metabolism and nitrogen excretion in lactating Holstein–Friesian cows [135]. Considering the role of these two genes in nitrogen utilization, albeit in a different species, and in metabolism and growth, they may be considered as potential candidate genes for protein efficiency in pigs.

The three overlapping RHM windows on SSC2 (30,144,793–32,170,603 bp) contained 16 (long) noncoding RNAs and, amongst others, the protein-coding genes *MPPED2*, *ARL14EP*, *FSHB*, *KCNA4,* and *METTL15*. In humans, *MPPED2* has been associated with kidney function [136] and, in pigs, it was included in a gene-co-expression module in muscle that differed between lean and obese breeds [137]. *KCNA4* has been reported in relation to production traits in pigs [138]. *METTL15* is required for mitochondrial protein synthesis [139] and has been linked to blood phosphorus levels in pigs [140]. Considering the reported functions of these genes, it is unclear whether they are plausible candidates for PE. It seems more likely that the noncoding RNAs in the highlighted region play a significant role in shaping the PE phenotype, although their targets remain to be identified.

The top-ranking variants for PE on SSC9 were located in intergenic regions (Table 2). We examined the combined region (2,013,927–2,812,088 bp) that included the overlapping top-ranked windows and extended 100 kb upstream and downstream of the variants (see Additional file 11, Table S11). In this region we identified several genes, including *PPFIBP2*, *OLFML1*, *SYT9*, *RBMXL2*, ENSSSCG00000060118 (largely overlapping with *RBMXL2*), *NLRP14*, LOC100524097, the transcription factor *ZNF215,* and a set of olfactory receptor genes. Some of these genes were linked to feed efficiency, muscle and adipose tissue metabolism, body weight, milk protein, immune function, and other relevant traits. *PPFIBP2*, involved in neuromuscular junction development (GO:0007528), was among the differentially expressed genes among Polish pig breeds [92] and upregulated in pale, soft, and exudative meat [141]. It was also differentially expressed in a non-alcoholic fatty liver pig model [142] and hypermethylated in the muscle of obese rabbits [143]. It has been proposed as a candidate gene for lactation persistency [144] and milk protein yield in buffalo [145], as well as for milk protein percentage [146] and carcass traits [147] in cattle. *SYT9,* which regulates dopamine secretion (GO:0014059) and exocytosis (GO:0070382 and GO:0017156, amongst others), shows maternal transmission ratio distortion in Entrepelado pigs, although the resulting phenotype remains unclear [148]. It was identified as a candidate gene for shear force in pork [130], for monounsaturated fatty acid content of the longissimus dorsi muscle in Ningxiang pigs [149], and for lactation persistency in buffalo [144].

*RBMXL2* has been proposed as a candidate gene for postnatal growth [150] and milk protein [146] and is contained in a region identified as selection signature linked to domestication in cattle [151]. In buffalo, it is associated with lactation persistency [144]. *NLRP14*, involved in the regulation of inflammatory response (GO: 0050727), has been associated with feed efficiency in cattle [152, 153]. It is highly expressed in the porcine intestine, but its predicted transcript in pigs contains multiple stop codons, indicating it may be an expressed pseudogene [154]. *ZNF215* is a well-known imprinted gene in vertebrates [155, 156] and has been identified as a candidate gene for shear force in pork [130] and for milk protein percentage in Holstein [155].

Olfactomedin proteins, such as *OLFML1*, are expressed throughout the brain, are key for the early development of the nervous system (neural crest), and are implicated in obesity [157] and non-alcoholic fatty liver disease [158, 159]. The region on SSC9 that was highlighted by both single-variant GWAS and RHM as potentially involved in PE contained 16 olfactory receptor genes. Olfactory transduction pathways and the olfactory receptor genes involved have been linked to residual feed intake in pigs [160], reflecting the extraordinary importance of olfaction in their foraging behaviour [161]. Olfactory pathways interact with satiety hormones like ghrelin, orexins, neuropeptide Y, insulin, leptin, and cholecystokinin [162]. In pigs, serum leptin correlated strongly with residual feed intake [163], with plasma leptin being significantly higher in high efficiency lines [164].

#### Mechanisms underlying feed intake, growth and efficiency

The mechanisms by which the identified genes influence ADFI, ADG, and PE range from direct effects, such as regulating feeding behavior (*QRFP*, *SORCS1*, *SYT9*, olfactory receptors) and nutrient sensing (*ASS1*), to indirect effects on immune function (*VAV2*, *BRD3*, *FIBCD1*, *ASS1*, *TRAF1*, *C5*), gut microbiome composition (*BRD3*, *FIBCD1*), and metabolism (*VAV2*, *LAMC3*, *ASS1*, *SORCS1*, *COL23A1*, *MPPED2*, *PPFIBP2*, *SYT9*, *OLFML1*, *MBD2*, *STARD6*, *RAB14*). Several genes have been linked to the urea cycle (*ASS1*) and protein utilization in dairy cattle (*COL23A1*, *PHYKPL*, *PPFIBP2*, *RBMXL2*, *ZNF215*), highlighting their relevance for improving PE and sustainability in livestock. In PigGTEx [68], considerable expression of these genes in relevant tissues (e.g. muscle, adipose tissue, brain, gastrointestinal tract), as well as a large number of molecular QTL and lncRNAs were documented. Apart from processes influencing gut microbiome composition and metabolism, pathways involved in nutrient sensing and regulation of feed intake may be of particular relevance to PE. Studies suggest that, if given the choice between diets that differ in protein content, pigs actively select a diet with a protein content that appeared to optimize their growth [165, 166] but the generalisation of these results and the exact underlying mechanisms remain unresolved.

Many of the highlighted genes overlap with a QTL on SSC1 for production traits in Swiss Large White pigs [33], including *ASS1*, *FIBCD1*, *LAMC3*, *QRFP*, and the lncRNA ENSSSCG00000042995, supporting their role as candidate genes for ADFI and related traits. However, another QTL identified by Noskova et al. [33] (at 157.7–162.7 Mb) was not detected in our study, likely due to differences in sample size, phenotyping, and diet composition. Noskova et al. [33] used deregressed progeny phenotypes with a ~ 5.5 × larger sample size, while our study measured feed intake directly for all animals on an experimental farm. Importantly, the diets also differed significantly: Noskova et al. [33] used a commercial diet optimized for performance, whereas the diets in the current study were formulated to mimic future sustainable feeding strategies by including more native protein feed crops. These diets also had a reduced crude protein and essential amino acid content (described in detail in [15]) and may have differed slightly in other aspects, such as digestible energy content (known to regulate feed intake in pigs [167]), fatty acid composition, and fiber content. Interestingly, *VAV2*, highlighted in our study for its numerous variants associated with ADFI, was not identified in the analysis of Noskova et al. [33].

Due to the limited sample size, we did not investigate pleiotropy among the traits, although the investigated traits are clearly interrelated and likely share some variants or QTL. Feed intake is an essential part of FCR and PE, and influences ADG. Theoretically, PE is a subset of FCR or overall feed efficiency, with the remaining portion largely associated with conversion of energy to adipose tissue. Consequently, fewer genes are expected to contribute to PE than to FCR, which should facilitate the detection of associated genes for PE compared to FCR, assuming that the distribution of genetic effects is similar for both traits. However, this may not be the case as the genetic architecture likely differs between PE and FCR, as suggested by our findings, which identified several plausible regions for PE but none for FCR. Furthermore, we would expect a non-zero negative genetic correlation between PE and FCR since the ability to convert dietary protein into muscle mass (PE) constitutes a component of the ability to convert feed into body mass (see [16] for a more detailed discussion). We previously reported a genetic correlation of −0.55 ± 0.14 between PE and FCR and of −0.53 ± 0.14 between PE and ADFI [15]. We could not find any overlapping regions associated with the respective traits in this study. Any overlapping regions may be below the thresholds and we thus failed to detect them. Moreover, as we have only detected a small number of genomic regions, the probability that these regions have an effect on both of these traits will be small. Additionally, none of the highlighted regions for PE on SSC2 and SSC9 matched entries for "feed efficiency" or "feed conversion ratio" in PigQTLdb using the JBrowse tool.

#### Challenges in variant detection for complex and difficult-to-measure traits

The limited ability to detect significant or suggestive associations in this study is likely due to the absence of large-effect SNPs and a relatively small sample size, both key factors for detecting genotype–phenotype associations. Although ADG, FCR, and lean meat content together currently account for only 18% of the dam line breeding goal (compared to 53% for the sire line), it is possible that genes with large effects on these traits have been fixed for the favourable alleles during selection history. Although PE has not yet been included in the breeding goal, it may have been indirectly improved through genetic correlations with ADFI, ADG, FCR or similar traits. According to Goddard and Hayes [168], detecting QTL with small effects requires large sample sizes. For this study, with around 1,000 animals and a genomic heritability of 0.42 for PE, only variants with effects > 5% of the phenotypic variance could be detected. Most variant effects here ranged from 0 to 1.4%, suggesting a sample size of at least 7,000 would be required to meet the Bonferroni threshold based on all variants.

Achieving the required sample size for PE is challenging due to the complexity of phenotyping this trait and the lack of high-throughput tools for efficient measurement. Models or estimation methods, such as approximating protein content from body weight, are commonly used in nutrition research, but assume that all pigs conform to an 'average' animal. This approach risks masking the individual variation in body composition among pigs of the same weight, which is critical for estimating breeding values and linking genomic to phenotypic variation [16]. Such inaccuracies and measurement errors can hinder QTL identification [169]. To address this, we used dual X-ray absorptiometry (DXA), which provides highly accurate PE phenotypes based on individual body composition measurements [44]. However, DXA is time consuming, requiring approximately 15 min per carcass, which poses a significant challenge for phenotyping large populations.

Our approach to compiling the phenotype data set may also have affected the statistical power to detect associated variants. To maximise sample size, we pooled data from different feeding trials with different dietary treatments (e.g. protein content), sex (entire males, females and castrated males) and age at slaughter, resulting in some level of heterogeneity. The genetic architecture of the traits likely varies across these factors. For example, sex differences in PE are well documented, with entire males having higher PE than females and castrated males [44, 46], although it remains unclear whether these differences depend on genotype (genotype-by-sex interactions). Genetic differences in PE across developmental stages are also suggested by genetic correlations below one and partially non-overlapping QTL [26, 27, 46]. Presence of genotype-by-feed interactions may influence results [16]. The polygenic architecture of PE suggests that, while some loci may be specific to certain age classes, sexes, or diets, others are likely consistent across these conditions, as demonstrated in [26]. In this study, we included covariates for systematic environmental effects (e.g., diet, age, sex) in the linear model, and used residuals for association testing. However, this adjustment only corrects for mean differences between groups and does not account for potential differences in genetic correlations across diets.

To identify more QTL in GWAS, we used whole-genome sequence data to increase the likelihood of including causal variants, which is expected to improve statistical power. However, as is the case with array data imputed to sequence level, this dramatically increases the number of variants and thus statistical tests, leading to a highly conservative genome-wide significance threshold. Additionally, because variants are not independent due to high LD, the number of tests is effectively inflated. One solution is to base the Bonferroni correction on the number of independent variants rather than the total number. Delpuech et al. [170] used the method by Gao et al. [171] to compute the number of independent SNPs and reduced ~ 570 K SNPs to 1690 independent variants, corresponding to a genome-wide threshold of −log10(P) = 4.5. However, with whole-genome data, this approach is computationally very expensive, as it requires computing correlation matrices between all variants per chromosome and then extracting the principal components needed to capture 99.6% of the genotype variability [171]. Instead, we adopted two alternative approaches: permutation testing [58, 59] and LD pruning based on published data on LD decay in commercial pig lines [62]. Permutation testing empirically generates the null distribution of the test statistic, allowing for the determination of an experiment-wide critical threshold. In our case, this resulted in −log_10_(P) values ranging from 7.35 to 7.50. LD pruning, which filters for independent variants (i.e., those below a defined LD threshold) based on typical species- or line-specific LD decay, yielded slightly less conservative thresholds of approximately −log10(P) = 7. However, this method does not rely on the specific data used in the study. For RHM, fewer tests are required, as thousands of variants are grouped into windows. For RHM, we also applied two thresholds, a classical Bonferroni correction (assuming windows are independent, although this may not always hold) and a threshold allowing one false negative per genome scan. These correspond to −log10(p) values of 4.7 (significant) and 3.4 (suggestive).

Another challenge with whole-genome data in RHM is inflated heritability estimates due to high LD of variants within windows. Since LD is unevenly distributed across the genome [62, 172], this can lead to an upward bias in genomic relatedness [173, 174]. Methods such as reweighting SNPs within a single GRM (LDAK) [175] or stratifying variants into LD and MAF bins across multiple GRMs (GREML-LDMS) [173] can reduce such biases in regional heritability estimates. We observed evidence of this issue in our data, albeit to a minor extent. The regional heritability estimates for the top 50 RHM windows in our study ranged mostly from 0.008 to 0.09 (Tables S5–S8), similar to findings in other studies [40, 176, 177]. For PE, regional heritabilities for the top 50 windows were generally low (0.01–0.06), except for one window on SSC9 (37500_42500) with a heritability of 0.12 ± 0.07. Notably, four overlapping windows for ADG on SSC14 had higher regional heritabilities (suggestive window 637,500_642.500, h^2^ = 0.15 ± 0.08; 640,000_645,000, h^2^ = 0.20 ± 0.09; 642,500_647,500, h^2^ = 0.24 ± 0.1; 645,000_650,000, h^2^ = 0.18 ± 0.09). Regional heritabilities for the top 50 windows for ADFI were also mostly low (0.02–0.08) except for two regions on SSC2 (375,000_380,000 and 380,000_385,000) with estimates of 0.30 ± 0.16 and 0.27 ± 0.13, respectively.

Higher marker density, such as whole-genome sequence data, improves genomic prediction accuracy only when combined with biological information, as shown in a simulation study [178]. Similarly, the inclusion of prior biological information can improve GWAS as it can aid in the detection of associated variants, for example through the use of Bayesian hierarchical models [179]. Useful prior information includes previously published QTL, genes and pathways relevant to the trait of interest, supported by detailed functional annotations [180], as well as data from various omics approaches and DNA accessibility studies. Molecular QTL, such as those for the expression of protein-coding genes, non-coding RNAs, enhancers and alternative splicing (e.g. pigGTEx project) [68], are particularly promising. Alternatively, biological information can be assigned to chromosomal regions (e.g. ~ 1 Mb) based on their gene content [170, 181]. In general, combining different methods and integrating existing resources can strengthen findings and provide additional evidence for associations that might otherwise fall below the significance threshold. This approach is particularly valuable for traits where phenotyping is challenging and sample sizes are inherently limited, ensuring optimal use of the available data.

## Conclusions

Here, we aimed to associate genomic variants with PE, a trait of particular interest for reducing the environmental impact of pork production through breeding, and other production traits. While single-variant GWAS and RHM analyses did not identify significant variants or windows for PE, we identified variants and genomic regions associated with ADFI and ADG, which are genetically correlated with PE. Several genes in these regions are plausible functional candidates for production traits and potentially for PE, including those involved in nutrient sensing, the urea cycle, and metabolic pathways, in particular IGF1-insulin. Notably, most variants highlighted by the single-variant GWAS for PE overlapped with top-ranked regions identified using RHM, providing further evidence for potential associations. While several of the highlighted genes have roles in nitrogen metabolism in cattle and in feed intake and muscle and adipose tissue metabolism in pigs, their specific roles in PE and production traits require further investigation. The role of gene regulation in shaping these traits, in particular the noncoding RNAs and their targets found in this study, should also be addressed in the future. The relatively small sample size, due to the challenges of measuring PE, likely limited the identification of significant variants. Nonetheless, despite the absence of major QTL, the traits showed considerable genomic heritability, suggesting a complex trait architecture with the contribution of numerous small-effect QTL. This genomic information could potentially be leveraged for genomic prediction. However, this requires a large reference population, emphasizing the need for faster phenotyping methods for PE. In summary, despite challenges associated with small sample sizes and difficult-to-measure traits, we identified plausible candidate genes for ADFI and ADG and highlighted potential candidates for PE through overlapping single-variant GWAS and RHM results. Development of more sensitive methods and further research into PE remains essential to realise its potential to improve the sustainability of pork production.

## Supplementary Information

Additional file 1: Table S1. Top 50 variants by GWAS for protein efficiency.

Additional file 2: Table S2. Top 50 variants by GWAS for average daily gain.

Additional file 3: Table S3. Top 50 variants by GWAS for average daily feed intake.

Additional file 4: Table S4. Top 50 variants by GWAS for feed conversion ratio.

Additional file 5: Table S5. Top 50 windows by RHM for protein efficiency.

Additional file 6: Table S6. Top 50 windows by RHM for average daily gain.

Additional file 7: Table S7. Top 50 windows by RHM for average daily feed intake.

Additional file 8: Table S8. Top 50 windows by RHM for feed conversion ratio.

Additional file 9: Table S9. Genes in two adjacent top-ranking windows in RHM for protein efficiency on SSC9 and in three overlapping top-ranking windows on SSC2.

Additional file 10: Table S10. Genes in two overlapping windows in RHM for average daily gain on SSC14 and in three overlapping top-ranking windows on SSC1.

Additional file 11: Table S11. Genes in the two overlapping windows in RHM that contained all significant and suggestive variants and the five windows in RHM for average daily feed intake on SSC1, which pass the suggestive threshold.

## Data Availability

A preprint of this manuscript has been deposited on bioRxiv (10.1101/2023.11.28.568963). Whole genome sequences for this study have been deposited in the European Nucleotide Archive (ENA) at EMBL-EBI under accession number PRJEB95768 (https://www.ebi.ac.uk/ena/browser/view/PRJEB95768). Phenotype is available on Zenodo (10.5281/zenodo.6985500).
